# The effect of dietary fat intake on hepatic gene expression in LG/J AND SM/J mice

**DOI:** 10.1186/1471-2164-15-99

**Published:** 2014-02-05

**Authors:** Charlyn G Partridge, Gloria L Fawcett, Bing Wang, Clay F Semenkovich, James M Cheverud

**Affiliations:** 1 Department of Anatomy and Neurobiology, Washington University in St. Louis, St. Louis, MO, USA; 2 Human Genome Sequencing Center, Department of Molecular and Human Genetics, Baylor College of Medicine, Houston, TX, USA; 3 Departments of Medicine and Cell Biology & Physiology, Washington University in St. Louis, St. Louis, MO, USA; 4 Department of Biology, University of Western Ontario, London, ON, Canada; 5 Department of Biology, University of Loyola Chicago, Chicago, IL, USA

**Keywords:** Liver, Dietary fat, Non-alcoholic fatty liver disease, NAFLD, Gene expression, Microarray, SM/J, LG/J

## Abstract

**Background:**

The liver plays a major role in regulating metabolic homeostasis and is vital for nutrient metabolism. Identifying the genetic factors regulating these processes could lead to a greater understanding of how liver function responds to a high-fat diet and how that response may influence susceptibilities to obesity and metabolic syndrome. In this study we examine differences in hepatic gene expression between the LG/J and SM/J inbred mouse strains and how gene expression in these strains is affected by high-fat diet. LG/J and SM/J are known to differ in their responses to a high-fat diet for a variety of obesity- and diabetes-related traits, with the SM/J strain exhibiting a stronger phenotypic response to diet.

**Results:**

Dietary intake had a significant effect on gene expression in both inbred lines. Genes up-regulated by a high-fat diet were involved in biological processes such as lipid and carbohydrate metabolism; protein and amino acid metabolic processes were down regulated on a high-fat diet. A total of 259 unique transcripts exhibited a significant diet-by-strain interaction. These genes tended to be associated with immune function. In addition, genes involved in biochemical processes related to non-alcoholic fatty liver disease (NAFLD) manifested different responses to diet between the two strains. For most of these genes, SM/J had a stronger response to the high-fat diet than LG/J.

**Conclusions:**

These data show that dietary fat impacts gene expression levels in SM/J relative to LG/J, with SM/J exhibiting a stronger response. This supports previous data showing that SM/J has a stronger phenotypic response to high-fat diet. Based upon these findings, we suggest that SM/J and its cross with the LG/J strain provide a good model for examining non-alcoholic fatty liver disease and its role in metabolic syndrome.

## Background

The relationship between dietary intake and metabolic syndrome is complex, with a number of genes, genetic interactions, and gene by environment interactions having significant effects on disease susceptibility and severity. Obesity-related metabolic disorders can occur when dietary energy intake chronically exceeds expenditure leading to a variety of conditions that include increased blood pressure, insulin resistance, and serum cholesterol levels [1]. Obesity *per se* is significantly influenced by both environmental factors, such as diet and exercise, and genetic factors, with heritability estimates ranging from 40% to 75% [2,3]. More critically, there is genetic variation among individuals in their responses to an obesogenic diet, with some being more likely to develop aspects of metabolic syndrome than others [4,5].

While metabolism involves a number of different organs, the liver is one of the key organs regulating nutrient homeostasis. Because of its direct involvement in dietary nutrient metabolism, the liver’s functional association to dietary obesity and metabolic syndrome is of keen interest. Previous work has shown that increased levels of dietary fat intake are associated with increased fat deposition in the liver and can lead to non-alcoholic fatty liver disease (NAFLD) [6-8]. This increase in hepatic fat is associated with a higher risk of obesity [9], insulin resistance [10-12], and type 2 diabetes mellitus [13,14]. As with obesity and metabolic syndrome, it has been suggested that susceptibility to NAFLD also has a strong genetic basis [15-19] with relatively high heritability values after controlling for age, sex, race, and body mass index [17]. Because of the strong association between hepatic fat accumulation and metabolic syndrome disorders, understanding how genetic factors influence the way in which the liver responds to increased dietary fat levels is critical.

Whole-genome expression studies have previously examined the effect of dietary fat intake on hepatic gene expression. A review of these studies shows that many of the genes whose expression is affected by dietary fat are related to lipid metabolism, adipocyte differentiation, defense against foreign bodies or injury, and stress response, particularly response to oxidative stress [20]. In a comprehensive study, Shockley et al. [21] examined hepatic gene expression profiles in relation to dietary fat and cholesterol for 10 different inbred mouse strains. Over all 10 strains, only Gene Ontology (GO) terms for cholesterol biosynthesis and isoprenoid metabolism were repressed by a high-fat diet in all of the strains. No biological GO terms were induced by a high-fat diet across all strains, indicating that differences in genetic background have a dominant effect on which genes and pathways respond to high dietary fat levels. However, the level of fat in the high-fat diet used by Shockley et al. [21] was modest (30% calories from fat), the low fat diet was not matched for other ingredients [22], and the dietary treatment lasted only from 6–10 weeks of age.

This study was designed to evaluate hepatic gene expression profiles for two mouse strains, LG/J and SM/J, on both a low and a high-fat diet. While the effect of dietary intake on hepatic gene expression has been assessed in a number of mouse strains, including SM/J [21], evaluating differences in expression levels between SM/J and LG/J provides a unique opportunity to examine the genetic factors associated with a number of obesity and metabolic syndrome related traits. LG/J and SM/J mice have been shown to differ in their response to a high-fat diet for traits involved in various metabolic syndrome domains [23,24]. SM/J individuals tend to be more responsive to the effect of a high-fat diet in relation to body weight, fat depot weight, organ weight, basal glucose levels, and triglyceride levels [23,24]. In addition, quantitative trait loci (QTLs) for a number of these phenotypes, including obesity [25-29], diabetes [25,30], serum lipid levels [25,31], fatty liver [32] and multiple domains of metabolic syndrome [33], have been mapped in populations derived from the intercross of these two strains. Thus, the goals of this project are twofold. First, we describe general differences in gene expression between these strains, between males and females, and between animals reared on low- and high-fat diets. More critically, we identify genes whose response to a high-fat diet differs between the LG/J and SM/J strains. Second, we relate these expression differences to genes located within previously defined QTLs where genetic effects were found to be diet-specific.

## Results

### Global gene expression

Of the 26,209 gene transcripts that showed significant expression levels, a total of 4,796 unique genes were differentially expressed among treatments. Of those, 3,880 transcripts were significantly different between SM/J and LG/J strains (Additional file 1), 1,224 were significantly different between males and females (Additional file 2), and 1,676 transcripts were significantly different by diet (Additional file 3). Three hundred transcripts showed a strain by diet interaction, 26 showed a significant strain by sex interaction, and only two showed a significant diet-by-sex-by-strain interaction, both of which corresponded to the gene *Cidea* (Table 1).@@@@

**Table 1:** Number of differentially expressed genes for each factor and their interactions

**Factor**	**Number of significant genes**
Diet	1676
Sex	1224
Strain	3880
Diet*Sex	26
Diet*Strain	300
Sex*Strain	0
Diet*Sex*Strain	2

*Strain Effects*. Forty-seven percent of the genes (1,840 gene transcripts) that were significantly different by strain were expressed more strongly in the LG/J strain and 53% more strongly in SM/J (2,040 gene transcripts) (Additional file 1). The genes that were significantly over-expressed within both LG/J and SM/J strains represented similar biological processes, such as lipid metabolism, protein metabolism and carbohydrate metabolic processes (Additional file 4). There were, however, differences in the biochemical pathways these genes represented (Additional file 4). Biochemical pathways that contained the largest number of genes over-expressed in SM/J included several response-to-stimulus pathways, including the platelet-derived growth factor (PDGF), integrin, and inflammation by chemokine and cytokine signaling pathways. In addition, a relatively large number of genes involved in apoptosis were over-expressed in SM/J. In LG/J, the metabolic-related pathways that were enriched included the purine metabolism and the cholesterol biosynthesis pathway. Other interesting pathways over-expressed in LG/J included the endothelin signaling pathway, the angiogensis pathway, and the insulin/IGF pathway.

*Sex Effects*. Of the 1,224 expressed transcripts that were significantly different between males and females, 46% were higher in males (568 gene transcripts) and 54% were higher in females (656 gene transcripts) (Additional file 2). As expected, genes that are sex-linked showed significant differences in expression between the sexes. Inactive X specific transcripts (*Xist*), located on the X chromosome, displayed the largest differences between males and females. Genes located on the Y chromosome, such as DEAD box polypeptide 3, Y-linked (*Ddx3y*), eukaryotic translation initiation factor 2, subunit 3, structural gene Y-linked (*Eif2s3y*) and ubiquitously transcribed tetratricopeptide repeat gene, Y chromosome (*Uty*), were expressed more in males. A number of cytochrome p-450 genes (*Cyp*) were also differentially expressed between the sexes (Additional file 2). Both males and females exhibited high expression in genes related to biological processes, such as lipid metabolism, carbohydrate metabolism, response to toxin, apoptosis and the generation of precursor metabolites and energy (Additional file 5), but the specific genes involved differed between males and females. On the other hand, the biochemical pathways represented by genes over-expressed in females differed substantially from those up-regulated in males. Pathways with genes expressed significantly higher in females were typically associated with amino acid biosynthesis, whereas pathways associated with increased expression in males were involved in a number of signaling pathways, including the heterotrimeric G-protein signaling pathway-Gi alpha and Gs alpha mediated pathway, the endothelial signaling pathway, and the cortocotropin releasing factor receptor signaling pathway (Additional file 5).

*Response to Diet*. For genes that were expressed differently on high and low-fat diets, 46% were higher in high-fat fed individuals (775 genes transcripts), while 54% (901 gene transcripts) were higher in low-fat fed individuals (Additional file 3). GO terms that were enriched for genes that showed significantly higher expression in high-fat fed individuals were involved in lipid and carbohydrate metabolism. Processes enriched in low-fat fed individuals included those involved in protein and amino acid metabolic processes (Additional file 6). Interesting biochemical pathways that were enriched by genes showing higher expression with high-fat diet are involved in stress response, such the p53 pathway, integrin signaling pathway, ubiquitin promeasome pathway, and the inflammation-mediated by chemokine and cytokine signaling pathways. Those enriched with genes that exhibited higher expression on a low-fat diet included blood coagulation, EGF receptor signaling, cholesterol biosynthesis, and a number of amino acid biosynthesis pathways (Additional file 6).

*Diet-by-Strain Interactions*. There were 259 genes (300 gene transcripts) whose expression exhibited a significant diet-by-strain interaction, i.e. diet affected expression differently in the two strains (Additional file 7). Gene expression was much more responsive to the high-fat diet in SM/J than in LG/J, with 95% of the diet related expression changes occurring in SM/J. GO terms enriched for genes exhibiting a diet-by-strain interaction included those involved in immune function, lipid and carbohydrate metabolic processes, and apoptosis (Table 2). The biochemical pathways that were significantly enriched included the plasminogen activating pathway, p53 pathway, angiogenesis, the integrin signaling pathway, and the blood coagulation pathway (Table 2).

**Table 2:** Biological processes and biochemical pathways enriched by genes exhibiting a diet by strain interaction

**GO annotation term**	**GO annotation**	**Mus musculus genes FEFLIST (26185)**	**Number of genes in pathway**	**Number of genes expected**	**Over/under represented**	**P-value**
Biological Process						
immune system process	GO:0002376	2974	62	20.22	+	1.72×10^-16^
antigen processing and presentation	GO:0019882	95	10	0.65	+	1.54×10^-9^
antigen processing and presentation of peptide or polysaccharide antigen via MHC class II	GO:0002504	35	6	0.24	+	1.90×10^-7^
response to stimulus	GO:0050896	2486	52	16.9	+	1.01×10^-13^
response to stress	GO:006950	547	13	3.72	+	1.08×10^-4^
cellular defense response	GO:0006968	564	16	3.83	+	1.93×10^-6^
response to toxin	GO:0009636	121	9	0.82	+	1.92×10^-7^
signal transduction	GO:0007165	4858	52	33.02	+	3.68×10^-4^
immune response	GO:0006955	900	16	6.12	+	4.75×10^-4^
Unclassified		10946	39	74.41	-	1.81×10^-8^
metabolic process	GO:0008152	9603	93	65.28	+	1.63×10^-5^
primary metabolic process	GO:0044238	9122	91	62.01	+	6.03×10^-6^
lipid metabolic process	GO:0006629	1266	24	8.61	+	6.06×10^-6^
carbohydrate metabolic process	GO:005975	1038	19	7.06	+	9.55×10^-5^
cellular process	GO:0009987	7133	69	48.49	+	5.51×10^-4^
endocytosis	GO:0006897	604	14	4.11	+	7.65×10^-5^
apoptosis	GO:0006915	1035	18	7.04	+	2.73×10^-4^
developmental process	GO:0032502	3296	38	22.41	+	7.37×10^-4^
system development	GO:0048731	2222	29	15.1	+	5.23×10^-4^
transport	GO:0006810	3009	36	20.45	+	5.33×10^-4^
**Biochemical Pathway**						
Plasminogen activation cascade		18	3	0.12	+	2.74×10^-4^
p53 pathway		127	5	0.86	+	1.89×10^-3^
Angiogenesis		193	6	1.31	+	2.22×10^-3^
Blood coagulation		55	3	0.37	+	6.52×10^-3^
Integrin signalling pathway		185	5	1.26	+	9.06×10^-3^
Androgen/estrogene/progesterone biosynthesis		28	2	0.19	+	1.59×10^-2^
Ornithine degradation		3	1	0.02	+	2.02×10^-2^
FAS signaling pathway		38	2	0.26	+	2.80×10^-2^
Methylmalonyl pathway		5	1	0.03	+	3.34×10^-2^
Xanthine and guanine salvage pathway		6	1	0.04	+	4.00×10^-2^
p53 pathway feedback loops 2		51	2	0.35	+	4.77×10^-2^

**Figure 1:**
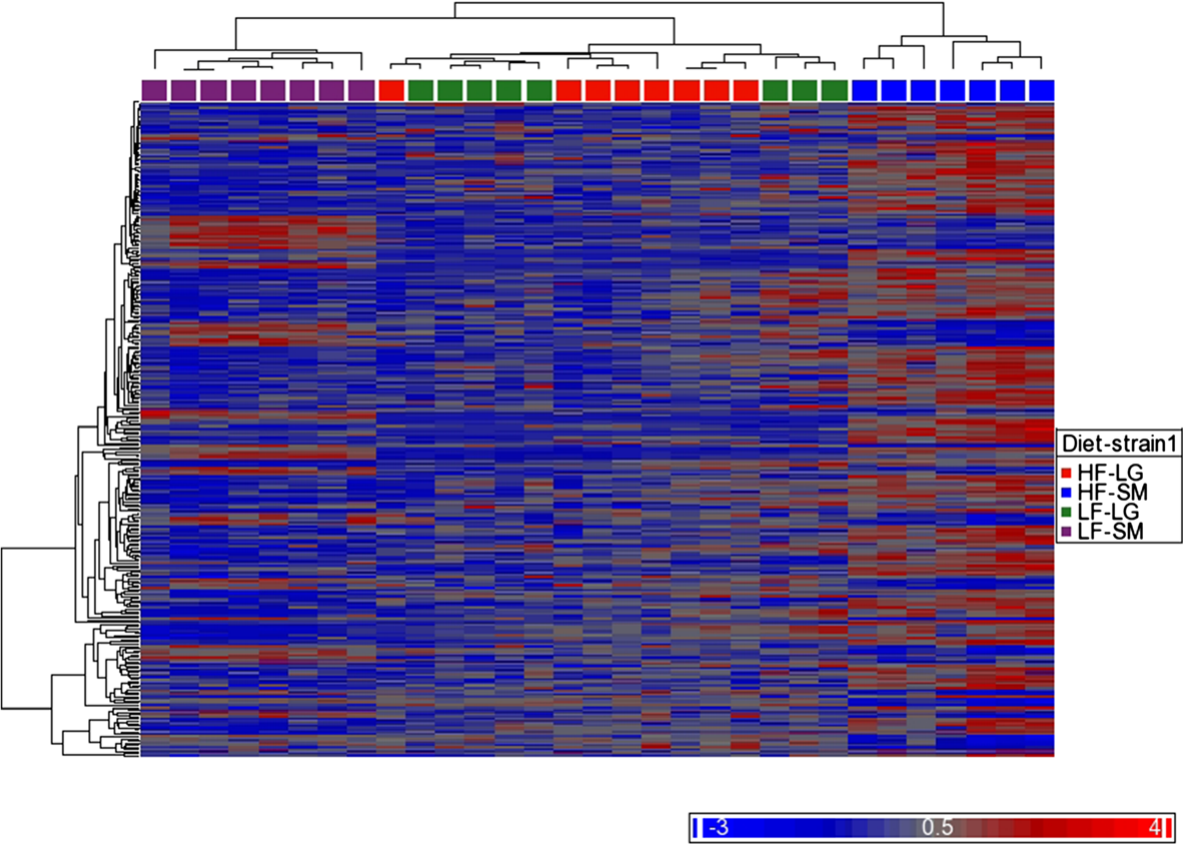
**Heat map of genes exhibiting a diet by strain interaction.** Hierarchical cluster analysis was performed producing two clusters, with the high-fat fed SM/J group clustering separately from all other groups. HF-LG: High-fat LG/J; HF-SM: High-fat SM/J; LF-LG: Low-fat LG/J; LF-SM: Low-fat SM/J.

In order to evaluate gene clusters that responded similarly for genes exhibiting a diet-by-strain interaction, a K-means clustering analysis was preformed and produced 2 stable gene clusters. Cluster 1 is composed of genes involved in response to stimulus pathways, such as the p53 pathway, integrin signaling pathway, and the TGF signaling pathway (Additional file 8). Cluster two was comprised of pathways related to multiple salvage pathways, the plaminogen activating cascade, and blood coagulation (Additonal file 8). Hierarchical biclustering of the data also show that high-fat fed SM/J individuals clustered separately from all other groups for these genes (Figure 1).

*Within Strain Effects.* Analyzing SM/J and LG/J separately for the effects of diet and sex on gene expression produced very different results. When SM/J was analyzed separately, diet had the strongest effect on gene expression with 2,137 gene transcripts being differentially expressed. Of these, 1,200 transcripts were up-regulated and 937 down-regulated on the high-fat diet (Additional file 9). Only 25 gene transcripts showed differential expression in relation to diet in the LG/J strain, 15 with increased expression and 10 with decreased expression on a high-fat diet (Additional file 9).

The major factor responsible for variation in LG/J gene expression was sex, with 637 transcripts differentially expressed between males and females. Of the 637 transcripts, 396 were higher in females and 241 were higher in males. This is in sharp contrast to the sex effects observed in SM/J, where only 190 transcripts showed differential expression (Additional file 10).

### Candidate Genes within QTLs

Studies of these strains have previously mapped QTLs affecting obesity, diabetes-related traits, and serum lipid levels in the LG,SM Advanced Intercross Lines (AIL). We correlated the list of genes in these QTLs with those demonstrating a differential response between strains to a high fat diet. Of the 259 genes showing different responses in LG/J and SM/J in relation to the high fat diet, 18 are located within QTLs previously mapped for serum lipid levels [31], 24 are located within QTLs mapped for obesity [27], and 20 are located within regions previous associated with diabetes [30] (Table 3). Chi-square analysis show QTLs previously identified as associated with serum lipid levels and obesity were significantly enriched with genes showing significant diet-by-strain interactions (Obesity: *χ*^2^=5.95, p=0.015; Lipids: *χ*^2^=6.91, p=0.008). However, QTLs associated with diabetes were not enriched (*χ*^2^=1.49; p=0.22). This result indicates that differential gene expression between strains in response to diet is the likely source of at least some of the mapped QTL effects.

**Table 3:** Genes transcripts with a significant diet by strain interaction for lipid, obesity, and diabetes QTLs

**QTL**	**Probe ID**	**Gene ID**	**Chromosome**	**Start**	**Stop**	**Strand**
*Lipid Serum QTLs*						
*Dserum1b*	ILMN_2717387	*Fbxo36*	1	84836416	84897062	+
*Dserum1b*	ILMN_2502317	*Ugt1a10*	1	89951963	90115579	+
*Dserum1b*	ILMN_2754718	*Ugt1a9*	1	89967375	90115572	+
*Dserum1c*	ILMN_2954575	*Arhgap30*	1	173319085	173340429	+
*Dserum3a*	ILMN_2529128	*LOC329702*	3	89169322	89178368	+
*Dserum3a*	ILMN_2883392	*S100a11*	3	93324410	93330209	+
*Dserum3a*	ILMN_1213457	*Snx27*	3	94301466	94386638	-
*Dserum3a*	ILMN_2837802	*BC028528*	3	95687877	95695928	-
*Dserum3a*	ILMN_2656422	*BC028528*	3	95687877	95695928	-
***Dserum4a***	**ILMN_2674367**	***Agrn***	**4**	**155539399**	**155560010**	**-**
***Dserum5a***	**ILMN_2690061**	***Hnrpdl***	**5**	**100462596**	**100468683**	**-**
*Dserum8a*	ILMN_2719473	*Asf1b*	8	86479406	86494096	+
***Dserum10a***	**ILMN_3100812**	***Gpx4***	**10**	**79509911**	**79519184**	**+**
***Dserum10a***	**ILMN_2684855**	***Gpx4***	**10**	**79509911**	**79519184**	**+**
*Dserum17a*	ILMN_2729447	*9030612M13Rik*	17	32910210	32924492	-
*Dserum17a*	ILMN_1236993	*March2*	17	33825041	33855598	-
*Dserum17a*	ILMN_1226525	*H2-Ab1*	17	34400185	34406355	+
*Dserum17a*	ILMN_2631423	*H2-Ab1*	17	34400185	34406355	+
*Dserum17a*	ILMN_2913716	*H2-Ab1*	17	3440185	34406355	+
*Dserum17a*	ILMN_1239102	*H2-Eb1*	17	34442843	34453144	+
*Dserum17a*	ILMN_2741935	*H2-Ea*	17	34479878	34481588	-
*Dserum17a*	ILMN_2599858	*Pbx2*	17	34729416	34734286	+
*Dserum18a*	ILMN_1226868	*Mapk4*	18	74088140	74225013	-
*Dserum19a*	ILMN_2677859	*Insl6*	19	29395844	29399808	-
*Dserum19a*	ILMN_2738893	*Ermp1*	19	29682704	29722905	-
*Obesity QTLs*						
*Dob2a*	ILMN_1220441	*Camk1d*	2	5214503	5635561	-
***Dob3a***	**ILMN_1219717**	***Sort1***	**3**	**108087009**	**108164429**	**+**
*Dob4a*	ILMN_247997	*2310040A07Rik/Enho*	4	41585177	41587357	-
*Dob6a*	ILMN_2656021	*Osbpl3*	6	50243329	50406200	-
*Dob6a*	ILMN_2974064	*Osbpl3*	6	50243329	50406200	-
*Dob6e*	ILMN_1221060	*Pparg*	6	115372091	115440419	+
*Dob6e*	ILMN_1216056	*2510049J12Rik*	6	115533562	115542594	-
*Dob6e*	ILMN_2956092	*Rassf4*	6	116583026	116623809	-
*Dob6e*	ILMN_2686244	*Rassf4*	6	116583026	116623809	-
*Dob6e*	ILMN_2672698	*Rassf4*	6	116583026	116623809	-
*Dob8a*	ILMN_2912598	*Ap3m2*	8	23897827	23916099	-
*Dob8c*	ILMN_2719473	*Asf1b*	8	86479406	86494096	+
***Dob10b***	**ILMN_1215807**	***Glipr1***	**10**	**111422511**	**111439687**	**-**
*Doc10c*	ILMN_3162796	*Cnot2*	10	115922222	116018557	-
*Doc10c*	ILMN_2878071	*Lyz*	10	116724853	116729924	-
*Dob13a*	ILMN_2865016	*Cd83*	13	43880572	43898501	+
*Dob14a*	ILMN_2627022	*Itih4*	14	31699662	31715167	+
*Dob14a*	ILMN_1231336	*Itih3*	14	31721762	31736731	-
*Dob17b*	ILMN_1236993	*March2*	17	33825041	33855598	-
*Dob17b*	ILMN_1226525	*H2-Ab1*	17	34400185	34406355	+
*Dob17b*	ILMN_2631423	*H2-Ab1*	17	34400185	34406355	+
*Dob17b*	ILMN_2913716	*H2-Ab1*	17	34400185	34406355	+
*Dob17b*	ILMN_1239102	*H2-Eb1*	17	34442843	34453144	+
*Dob17b*	ILMN_2741935	*H2-Ea*	17	34479878	34481588	-
*Dob17b*	ILMN_2599858	*Pbx2*	17	34729416	34734286	+
*Dob17b*	ILMN_2665266	*H2-T10*	17	36254035	36258389	-
*Dob17b*	ILMN_1230878	*H2-T10*	17	36254035	36258389	-
*Dob17b*	ILMN_2783997	*Trim10*	17	37006537	37014750	+
*Dob17b*	ILMN_2426853	*Ubd*	17	37330873	37332782	+
*Dob17b*	ILMN_2964185	*H2-M2*	17	37617796	37620474	-
*Dob17b*	ILMN_2742311	*Cyp39a1*	17	43804474	4388794	+
*Dob19a*	ILMN_1230587	*Lpxn*	19	12873133	12908301	+
*Diabetes QTLs*						
*Ddiab3a*	ILMN_1222860	*381484*	3	15848070	15906332	-
*Ddiab4a*	ILMN_2773215	*Epb4.1l4b*	4	57004844	57156309	-
*Ddiab4a*	ILMN_2775064	*Epb4.1l4b*	4	57004844	57156309	-
*Ddiab4b*	ILMN_2736168	*Ppt1*	4	122513485	122536418	+
*Ddiab5a*	ILMN_2424721	*Pdgfa*	5	139451968	139473324	-
*Ddiab6a*	ILMN_2656021	*Ospl3*	6	50243329	50406200	-
*Ddiab6a*	ILMN_2974064	*Osbpl3*	6	50243329	50406200	-
*Ddiab5d*	ILMN_1221060	*Pparg*	6	115372091	115440419	+
*Ddiab5d*	ILMN_1216056	*2510049J12Rik*	6	115533562	115542594	-
*Ddiab7b*	ILMN_2658804	*Rras*	7	52273348	52277016	+
*Ddiab7c*	ILMN_2707494	*Mcee*	7	71537531	71557007	+
*Ddiab8a*	ILMN_2912598	*Ap3m2*	8	23897827	23916099	-
*Diab8b*	ILMN_2767918	*Ifi30*	8	73286673	73290562	-
*Diab8b*	ILMN_1228213	*Ifi30*	8	73286673	73290562	-
*Diab8b*	ILMN_2749747	*Haus8*	8	73772460	73796833	-
*Ddiab11a*	ILMN_2727503	*Igfbp3*	11	7106089	7113900	-
***Diab11d***	**ILMN_3147074**	***Pecam1***	**11**	**106515531**	**106611942**	**-**
*Ddiab13a*	ILMN_2595395	*Slc17a2*	13	23898862	23917049	+
*Ddiab13a*	ILMN_1217058	*Slc17a2*	13	23898862	23917049	+
*Ddiab13c*	ILMN_3122081	*5133401N09Rik*	13	58259015	58266052	+
*Ddiab14b*	ILMN_2627022	*Itih4*	14	31699662	31715167	+
*Ddiab14b*	ILMN_1231336	*Itih3*	14	31721762	31736731	-
*Diab15b*	ILMN_2543688	*Snord123*	15	32170324	32176484	
*Ddiab16a*	ILMN_1222821	*Rogdi*	16	5008823	5013610	-
***Ddiab17a***	**ILMN_2933463**	***Plg***	**17**	**12571474**	**12612250**	**+**
*Ddiab19a*	ILMN_2744398	*Ostf1*	19	18653818	18706279	-

Transcripts in bold are located just outside the 95% CI for the QTL and were not included in the enrichment analysis.

## Discussion

This study has two main goals. The first, to characterize hepatic gene expression profiles for SM/J and LG/J inbred mouse strains and examine how these profiles were influenced by diet and sex. The second goal is to examine how expression profiles differ between these two strains in relation to diet, and to characterize potential candidate genes located within previously mapped QTLs that are associated with traits from metabolic syndrome domains. As expected, our results show that diet, sex, and strain all have significant impacts on gene expression, with many genes showing strain-specific dietary responses for gene expression.

Overall, the impact of the high-fat diet on gene expression appears to be similar to that found in other expression studies [20]. There are many more effects than noted in Shockley et al. [21] perhaps because of the higher amount of fat in the diet utilized here (42% vs 30%) and the prolongation of the dietary treatment, from 6–10 weeks compared to 3–20 weeks in our study. A number of defense and stress response pathways were enriched by genes over-expressed in high-fat fed mice, including the p53, the inflammation mediated by chemokine and cytokine, and the ubiquitin signaling pathways, which regulate cellular damage response and influence inflammatory response. Enrichment of these pathways supports previous work suggesting that a high-fat diet can lead to cellular oxidative stress and increased inflammation within the liver, potentially resulting in nonalcoholic steatohepatitis (NASH), liver fibrosis, and further exacerbating insulin resistance [34-38].

SM/J and LG/J differ in their phenotypic response to a high-fat diet, with SM/J being more responsive than LG/J for many obesity, diabetes-related, and serum lipid level traits [23,24]. While these phenotypic differences between these strains have previously been observed, the stark differences in how diet impacts gene expression is somewhat surprising. A total of 259 unique genes displayed significant diet-by-strain interactions. In 95% of these cases, SM/J mice displayed a greater change in expression in relation to diet than LG/J mice. Hierarchical cluster analysis of these genes grouped high-fat fed SM/J separately from all other groups (Figure 1), again demonstrating that SM/J is more responsive to dietary fat intake than LG/J. This suggests that genetic background plays a significant role in influencing how genes respond to high-fat diet.

Similar to these results, Shockey et al’s. [21] assessment of how high fat diet affected gene expression showed that only a few biochemical pathways were commonly affected across the strains that were analyzed. This suggests that there is little consistency between strains in what is up- or down-regulated on a high fat diet. This is an important consideration because it means that genetic background plays a significant role in how diet impacts gene expression in this system. Thus, one cannot characterize a general murine response to a high fat diet for hepatic expression using any specific strain, such as C57BL/6J. While this may seem like an obstacle for murine studies of hepatic gene expression, it also provides a great opportunity to examine how genetic background influences these effects.

The biological processes that are most enriched with genes showing diet-by-strain interactions were mainly related to immune system processes, specifically antigen processing and presentation. Most of the differences were driven by the effects of the high-fat diet in SM/J mice. Associated with these are a number of transcripts involving major histocompatibilty complexes (MHC) I and II (*Hfe, H2-D1, H2-D4, H2-Ea, H2-Ab1, H2-DMa*) and killer cell activation (*Tyrobp, Pira11*). In most cases, there was a significant increase in the expression of these genes with a high-fat diet in SM/J and either no change or a slight decrease in expression in LG/J. This suggests a heightened immune response in SM/J with a high-fat diet, compared to LG/J mice. There is substantial evidence suggesting a strong association between immune response and metabolic function [39]. In particularly, diets that are high in fat have been shown to trigger immune response, particularly through inflammation in a number of different tissues (including adipose and liver) [40-42]. For example, MHC-II expression tends to increase when cells are under oxidative stress [43,44], and increased expression of MHC-II associated genes in hepatic cells can be induced by altering levels of dietary cholesterol [45]. Severe oxidative stress can lead to cellular damage, resulting in further hepatic inflammation, and potentially the development of heptatic steatosis and insulin resistance [34-38]. The difference in immune response in relation to diet between these two strains may, in part, explain why they differ in their response to dietary treatment. In particular, it provides some information as to why SM/J mice may show diminished glucose tolerance in comparison to the LG/J strain [24] on a high fat diet.

Biochemical pathways that were enriched with genes showing a diet-by-strain interaction included the plasminogen activating pathway, p53 pathway, and angiogenesis (Table 2). As a consequence of increased hepatic inflammation and hypoxia, which are associated with immune response, angiogenesis is commonly induced in order to increase blood flow and provide oxygen and nutrients to damaged areas [46,47]. Interestingly, Liu et al. [48] found that angiogenesis in a skin wound-healing model was higher in the MRL strain, which shares 75% of its DNA identical-by-descent with LG/J, than in other strains. In our data, we found that diet affected many genes within this pathway differently and in a strain-specific fashion. Some genes, including angiopotin (*Ang)* and mitogen-activated protein kinase 4 (*Mapk4)*, showed decreased expression in high-fat fed SM/J mice, Rous sarcoma oncogene (*Src)* and *Mapk4* showed increased expression in high-fat fed LG/J mice and the remaining genes in this pathway with a diet-by-strain effect (i.e., platelet derived growth factor (*Pdgfa)*, docking protein 2 (*Dok2)* and leupaxin (*Lpxn)*) exhibited increased expression in high-fat fed SM/J (Table 4). Still, the actual phenotypic effect that these gene expression changes have on the liver is unclear. For example, *Ang* and *Pdgfa*, along with other growth factors, have a strong pro-angiogenic effect when up-regulated [47]. In our data gene expression levels for *Ang* were lower in SM/J animals fed a high-fat diet, while *Pdgfa* expression levels significantly increased. Similarly, *Lpxn* encodes for the protein leupaxin, a member of the paxillin protein family, which play an important role in focal cell adhesion organization and signal transduction within the extracellular matrix [49,50]. It also provides a platform for SRC protein binding [50,51]. In our data set *Src* showed increased expression on a high-fat diet in LG/J, while *Lpxn* only showed increased expression in high-fat fed SM/J animals. It, therefore, appears that while high-fat diet does impact the angiogenesis pathway for both LG/J and SM/J, the genes that are impacted differ between the two. To clarify these results future studies assessing the effects of high-fat diet on angiogenesis for these strains should continue. Future studies would also be interesting considering how closely related LG/J is to MRL and MRL’s strong healing phenotype.

**Table 4:** Genes expressing significant diet-by-strain interactions within enriched biochemical pathways

**Biochemical pathways**	**Genes within pathway**
Plasminogen activating cascade	Serpinf2 (↓HFSm), Plg^*D*^*(*↓HFSm), Fga(↓HFSm)
p53 pathway	Igfbp3^*D*^ (↑HFSm), E2f1 (↑HFSm), Ccng1(↑HFSm), Tnfrsf6(↑HFSm), Cdc2a(↑HFSm)
Angiogenesis	Src(↑HFLg), Ang(↓HFSm), Mapk4^*L*^(↑HFLg, ↓HFSm), Dok2^*FL*^(↑HFSm), Lpxn^*MS,Ob*^(↑HFSm), Pdgfa^D^(↑HFSm)
Blood coagulation	Serpinf2(↓HFSm), Plg^*D*^(↓HFSm), Fga(↓HFSm)
Integrin signalling pathway	Fn1(↑HFLg, ↓HFSm), Src(↑HFLg), Rras^*D*^(↑HFSm), Mapk4^*L*^(↑HFLg, ↓HFSm), Arl11^*FL*^(↑HFSm)
Androgen/estrogene/progesterone biosynthesis	Hsd3b4(↓HFLg), Hsd3b2(↓HFSm)
Ornithine degradation	Azi2(↓HFSm)
FAS signaling pathway	Capg(↑HFSm), Tnfrsf6(↑HFSm)
Methylmalonyl pathway	Mcee^*MS,P*^(↑HFSm)
Xanthine and guanine salvage pathway	Hprt1(↑HFSm)
p53 pathway feedback loops 2	E2f1(↑HFSm), Ccng1(↑HFSm)

Arrows indicate wether expression levels were higher or lower in high-fat fed individuals verses low-fat fed individuals. HFSm=High-fat fed SM/J, HFLg=High-fat fed LG/J. Genes with superscripts are located within and near previously identified QTLs for a number of traits.

^*D*^*Diabetes QTL,*^*L*^*Lipid QTL,*^*FL*^*Fatty Liver QTL,*^*MS*^*Metabolic Syndrome QTL,*^*O*^*Obesity QTL.*

One of the most interesting aspects of this study is that QTLs associated with many metabolic syndrome domains, including obesity, diabetes, cholesterol and triglyceride levels, and fatty liver, have previously been mapped in LG/J by SM/J crosses using the same high and low-fat diets as used in this study [25,27,30-33]. Thus, by examining how gene expression profiles differ between these strains, particularly for genes within these QTL regions, we can narrow the number of positional candidate genes influencing individual dietary response. Of the 259 gene transcripts with a significant diet by strain interaction, 57 were located within previously mapped QTLs. There was a significant enrichment of genes for obesity and serum lipid level QTLs, although, no such enrichment occurred for diabetes-related QTLs. Several metabolic syndrome components are associated with genes within and around these regions including peroxisome proliferator-activated receptor gamma (*Pparg*), energy homeostasis (*Enho*), insulin-like growth factor binding protein 3 (*Igfbp3*), palmitoyl-protein thioesterase 1 (*Ppt1*), and sortilin 1 (*Sort1*).

**Figure 2:**
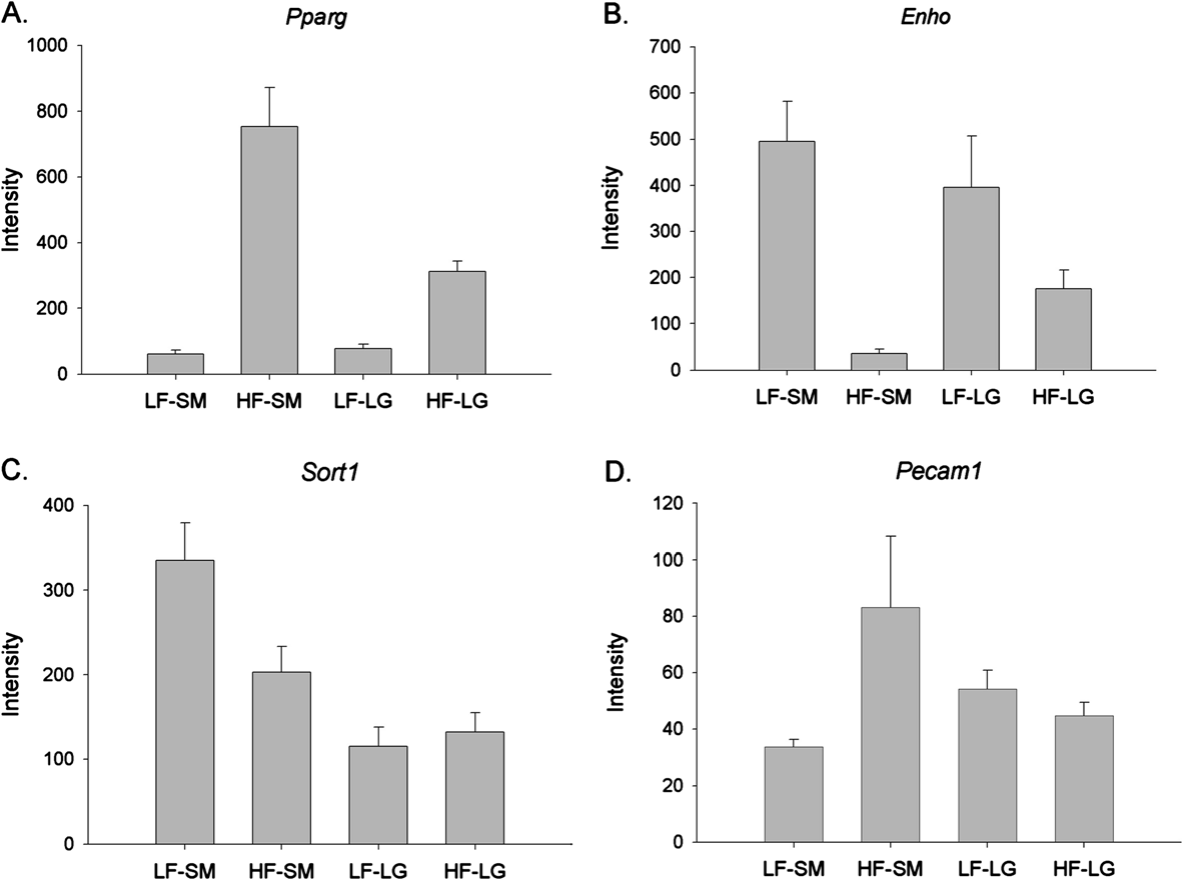
**Least square mean intensity values for genes within or near previously identified QTLs that exhibited a diet by strain interaction. (A)** Peroxisome proliferator-activated receptor gamma (*Pparg*); **(B)** Energy homeostasis (*Enho*); **(C)** Sortilin (*Sort1*); **(D)** Platelet/endothelial cell adhesion molecule 1 (*Pecam1*). Abbrev: LF-SM: Low-fat SM/J; HF-SM: High-fat SM/J; LF-LG: Low-fat LG/J; HF-LG: High-fat LG/J. Graphs based on LS mean of raw intensity data. Error bars represent standard error of the mean.

Genes associated with lipid metabolism, such as *Pparg* and *Enho*, were differentially expressed between SM/J and LG/J strains. *Pparg* is well known as a major factor involved in dietary obesity and diabetes [52-54] and one of the major regulators of adipocyte differentiation. This gene is located within a QTL affecting both obesity- [27] and diabetes-related traits [30]. Gene expression profiles for *Pparg* showed that high-fat diet increased expression of this gene in both SM/J and LG/J, although this increase was significantly greater in SM/J individuals (Figure 2A). *Enho* produces adropin, a protein involved in glucose and lipid homeostasis [55], and is located within a QTL associated with obesity [56]. Increased levels of adropin in transgenic mice were found to be associated with improved response to diet-induced obesity, insulin resistance, and glucose tolerance [54]. In SM/J, the high-fat diet significantly reduces the expression of *Enho,* promoting obesity. Expression was also reduced in high-fat fed LG/J animals, but this decrease was not as severe as that observed in SM/J (Figure 2B).

Sortilin (*Sort1*) plays a significant role in the release of low-density lipoprotein (LDL) cholesterol from the liver to the blood stream making it a potentially important gene for NAFLD and is located just outside the 1-LOD drop support interval of a QTL mapped for obesity [27]. Recently, single nucleotide polymorphisms (SNPs) located in an enhancer region of this gene have shown to be associated with an increased risk of myocardial infarction and increased LDL cholesterol levels. In this study, we found that the overall levels of *Sort1* were significantly higher in SM/J than in LG/J. However, high-fat diet significantly lowered expression of *Sort1* in SM/J, while this effect was not observed for the LG/J strain (Figure 2C). Similarly, total cholesterol levels from blood serum tend to be lower in SM/J compared to LG/J, but these values respond more to diet in SM/J, with high-fat diet eliciting a significant increase [24].

Genes that have been shown to impact NAFLD were also differentially expressed between LG/J and SM/J. Platelet/endothelial cell adhesion molecule 1 (*Pecam1*) is a glycoprotein located near a QTL for fatty liver [32] and diabetes [30]. Previous work has suggested that *Pecam1* is involved in regulating inflammation and higher expression of this gene protects the liver from the effect of high dietary fat and NAFLD [57]. High-fat fed SM/J displayed significantly higher *Pecam1* expression levels when compared to low-fat fed individuals. The effect of diet on *Pecam1* expression was not significant in LG/J (Figure 2D). This may suggest that the liver in SM/J mice is under increased stress when fed a high-fat diet relative to LG/J. Overall, the expression profiles of genes in QTLs on a high fat diet, support increased obesity, serum lipid levels, and diabetes associated with the SM/J alleles.

**Figure 3:**
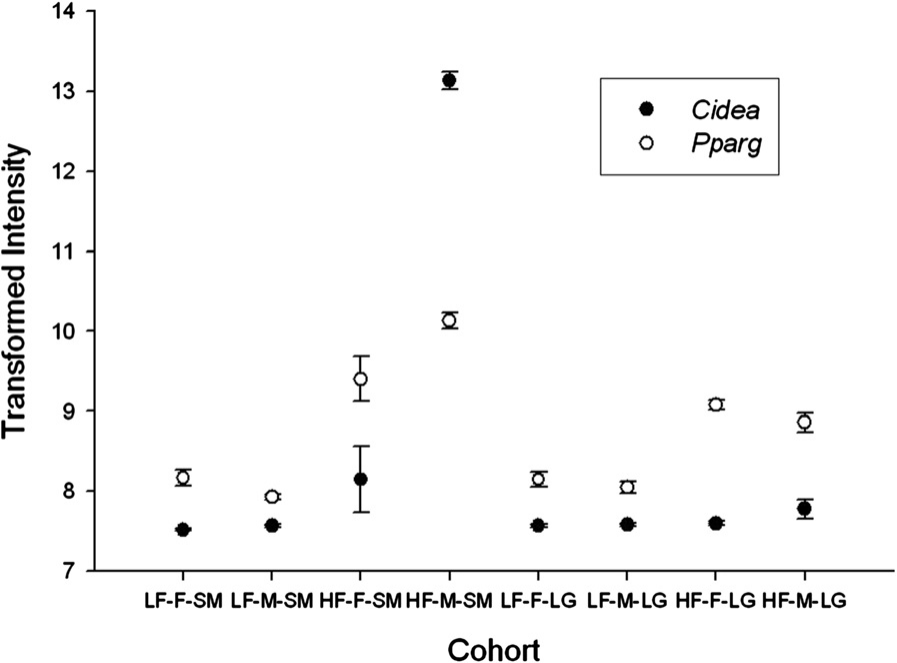
**Comparison of *Cidea *and *Pparg *intensity values.** Abbrev: LF-F: Low-fat female; LF-M: Low-fat male; HF-F: High-fat female; HF-M: High-fat male. Transformed data are used for comparison purposes. Error bars represent standard error of the mean.

The only gene transcripts exhibiting a significant 3-way diet-by-sex-by-strain interaction were for the cell death-inducing DNA fragmentation factor-alpha (*Cidea*) gene. While this gene is not located within a previously identified QTL, mice deficient in *Cidea* do show increased metabolic rates and resistance to obesity when on a high-fat diet [58]. *Cidea* expression is strongly associated with the production of lipid droplets in white adipose tissue, with increased expression enhancing the size of the lipid droplets [59]. In addition, this increase is also associated with increased insulin sensitivity [59]. *Cidea* expression is typically regulated by *Pparg* and our data show that *Cidea* and *Pparg* show similar expression trends (Figure 3). However, the 3-way interaction of *Pparg* was not significant at the whole genome level. Remaining questions are why such strong differences in *Cidea* expression were observed between SM/J males and SM/J females and whether or not these effects are also observed in other strains. To our knowledge, there is no data within the literature addressing sex specific differences in *Cidea* expression in relation to diet. Most studies examining expression levels do not separate out sex effects, with few exceptions [60], and indeed, for our data set, the effect observed in SM/J males were strong enough that when SM/J males and SM/J females were pooled together a significant increase in *Cidea* was still observed. Thus, future studies would benefit from separating out and examining the sexes individually in order to determine if significant sex effects are likewise occurring.

## Conclusions

These data show that dietary fat intake significantly impacts gene expression levels, particularly in SM/J relative to LG/J. This is consistent with previous phenotypic data that has shown SM/J to be more responsive than LG/J to a high-fat diet for metabolic syndrome associated traits, such as obesity, diabetes and lipid serum levels [23,24]. Many of the genes that are affected by diet are related to cellular defense, stress, and inflammation, suggesting that increased dietary fat intake promotes processes related to hepatic inflammation more so in SM/J than in LG/J. As the prevalence of NAFLD and its association with obesity and increased risk for metabolic syndrome steadily increases among Western societies, it is becoming vital that we understand the factors that contribute to individual variability in and susceptibility to this disease. Previous work has shown that genetic differences between SM/J and LG/J contribute to the amount of fat that accumulates within the liver on a high-fat diet [32] and QTLs for this trait have been mapped in recombinant inbred strains produced from these two parent strains. In accordance with this and our findings that gene expression profiles in response to high-fat diet produces a strong inflammatory response within the liver, particularly for SM/J, we suggest that SM/J and its cross with the unresponsive LG/J strain are a good model for examining non-alcoholic fatty liver disease and its role in the metabolic syndrome.

## Methods

In order to assess the effects of dietary intake on gene expression in SM/J and LG/J mice, males and females from each strain were placed on either a low-fat diet (15%, Research Diets #D12284) or high-fat (42%, Harlan-Teklad #TD88137) diet immediately after weaning (3weeks) until 20 weeks of age (Table 5). Previous work has shown that SM/J mice consume more calories per gram of body mass than LG/J; however, there is no difference in the amount of energy consumed per body mass between individuals fed a high-fat versus a low-fat diet for either strain [24]. At 20 weeks, mice were sacrificed in late morning after a four-hour fast and tissue was collected from 4 males and 4 females from each strain and diet. The liver tissue was immediately frozen in liquid nitrogen and stored at −80°C until extraction. All animal procedures were approved by the Washington University in St. Louis Institutional Animal Care and Use Committee (IACUC).

**Table 5:** Components of high-fat and low-fat diets

	**High fat diet**	**Low fat diet**
Energy from fat (%)	42	15
Casein (g/kg)	195	197
Sugars (g/kg)	341	307
Corn starch (g/kg)	150	313
Cellulose (g/kg)	50	50
Corn oil (g/kg)	0	58
Hydrogenated coconut oil (g/kg)	0	7
Anhydrous milk fat (g/kg)	210	0
Cholesterol (g/kg)	1.5	0
Kilojoules per gram	18.95	16.99

Total RNA was extracted using RNeasy® 96 Universal Tissue extraction kits (Qiagen, Valencia, CA) and quantified using a Nanodrop™ 2000 (Thermo Scientific, Wilmington, DE). RNA samples were submitted to the Washington University Microarray Core Facility, where quality was assessed using a 2100 Bioanalyzer (Agilent Tecnologies, Palo Alto, CA). RNA was reverse transcribed and amplified using an Illumina TotalPrep amplification kit (Ambion, Austin, TX) and then hybridized onto Illumina® WG-6 v.2 BeadChips (Illumina, San Diego, CA). Arrays were scanned using the Illumina Beadstation 500. Images were processed using Illumina BeadScan software and intensity values were analyzed using Illumina BeadStudio.

Illumina raw data from 45,281 unique probes were read into R statistical software using the Lumi package [61]. Data were transformed using a variance stabilization transformation [62], which takes into account the large number of technical replicates on Illumina arrays, and normalized using a robust spline normalization. Genes that showed no significant expression were filtered from the data set prior to analysis, leaving 26,209 transcripts analyzed for the liver. The data was then read into Partek Genomics software v. 6.5 (Partek Incorporated, St. Louis, MO) for further statistical analysis. An ANOVA was used to examine the impact of diet, strain, sex, and their interactions (diet x strain, diet x sex, sex x strain, and diet x sex x strain) on differential gene expression. A genome-wide false discovery rate threshold of 0.05 was used to determine statistical significance.

K-means and hierarchical clustering analysis were performed in Partek (Partek Incorporated, St. Louis, MO) on genes that showed significant diet-by-strain interactions (FDR<0.05). K-means clustering was preformed using a Euclidian distance function with 1,000 iterations utilizing different values of K. The number of clusters with the lowest David-Bouldin value was identified as the most likely value of K. Hierarchical clustering was performed on group mean data using a Euclidian distance function and average linkage method.

The Gene Ontology (GO) database *PANTHER* (http://pantherdb.org) was used to determine biological processes and biochemical pathways that were enriched with differentially expressed genes. Pathways and processes with p values<0.05 were considered to be significant.

In order to determine if previously identified QTLs for diabetes, serum lipid levels, and obesity were significantly enriched with genes exhibiting a significant diet-by-strain interaction, a chi-square test was used to determine if the number of these genes located within QTLs significantly differed from what would be expected by chance. Probability values of <0.05 were used to determine significance.

### Availability of supporting data

The data set supporting the results of this article are available in the ArrayExpress database (http://www.ebi.ac.uk/arrayexpress) under the accession number #E-MTAB-2172.

## Abbreviations

NAFLD: Non-alcoholic fatty liver disease; QTL: Quantitative trait loci.

## Competing interests

No conflicts of interest (financial or otherwise) are declared by the authors.

## Authors’ contributions

JMC conceived the study. CP, GF, and BW were involved in sample and data collection. CP performed the statistical analyses. CP, CFS, and JMC were involved in data interpretation. CP and JMC wrote the manuscript. All authors read and edited the manuscript.

## Supplementary Material

Additional file 1 Gene transcripts that are significantly different between SM/J and LG/J lines. Click here for file

Additional file 2 Gene transcripts that are significantly different between females and males. Click here for file

Additional file 3 Gene transcripts that are significantly different between diets. Click here for file

Additional file 4 Biological and biochemical processes enriched by genes exhibiting higher expression in either SM/J or LG/J. Click here for file

Additional file 5 Biological and biochemical processes enriched by genes exhibiting higher expression in either females or males. Click here for file

Additional file 6 Biological and biochemical process enriched by genes exhibiting higher expression with either low-fat or high-fat diets. Click here for file

Additional file 7 Genes that exhibited a significant diet-by-strain interaction. Click here for file

Additional file 8 Biological and biochemical process enriched by genes from cluster 1 and cluster 2. Click here for file

Additional file 9 Gene transcripts displaying differential expression with diet in SM/J. Click here for file

Additional file 10 Gene transcripts differentially expressed between the sexes in LG/J. Click here for file
